# Supplementary material for: Exploration of Bromodomain Proteins as Drug Targets for Niemann–Pick Type C Disease
Source: Int J Mol Sci. 2025 Jun 16;26(12):5769. doi: 10.3390/ijms26125769 (PMC12192928; doi:10.3390/ijms26125769)
Supplement: Supplementary file 1 [file ijms-26-05769-s001.zip › ijms-3624274-supplementary.pdf]

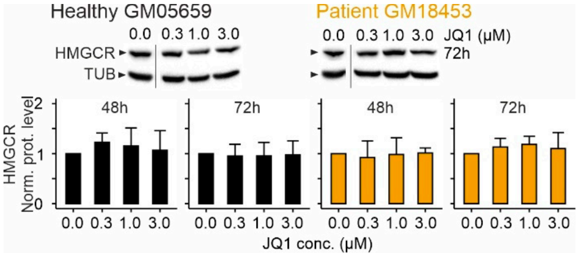

Figure S1. No effect of JQ1 on HMGCR levels in cultured human skin fibroblasts. Mean levels of 3-Hydroxy-3-Methylglutaryl-CoA Reductase (HMGCR) in primary cultures of skin fibroblasts from a healthy donor (GM05659; black) and a NPCD patient (GM018453; orange) after treatment with JQ1 or vehicle (DMSO) for indicated periods and at indicated concentrations (n = 4 preparations). Values were first normalized to tubulin (TUB) levels as loading control and then to vehicle-treated (JQ1 concentration zero) control cultures, respectively. Grayscale images show bands of HMGCR and of TUB from of representative immunoblots of indicated lysates. Note that for technical reasons the lanes corresponding to vehicle controls were run on the same filter but not continuous with the other lanes.
